# Recognizing Tumor Origin for Lymphoid Tumor of Unknown Primary *via* Total-Body PET/CT Scan—Case Report

**DOI:** 10.3389/fonc.2022.766490

**Published:** 2022-02-03

**Authors:** Weizhao Lu, Jianfeng Qiu, Xue Xie, Kun Li, Yanhua Duan, Min Li, Chao Ma, Zhaoping Cheng, Sijin Liu

**Affiliations:** ^1^Science and Technology Innovation Center, Shandong First Medical University & Shandong Academy of Medical Sciences, Jinan, China; ^2^Department of Radiology, Shandong First Medical University & Shandong Academy of Medical Sciences, Taian, China; ^3^Department of PET-CT, The First Affiliated Hospital of Shandong First Medical University, Shandong Provincial Qianfoshan Hospital Affiliated to Shandong University, Jinan, China; ^4^State Key Laboratory of Environment Chemistry and Ecotoxicology, Research Center for Eco-Environment Sciences, Chinese Academy of Sciences, Beijing, China

**Keywords:** cancer of unknown primary, metastasis, tumor detection, cancer imaging, total-body PET/CT

## Abstract

Localizing the site of tumor origin for patients with lymphoid tumor is fairly difficult before the definitive detection of the primary tumor, which causes redundant imaging examinations and medical costs. To circumvent this obstacle, the emergence of the world’s first total-body positron emission tomography/computed tomography (PET/CT) provides a transformative platform for simultaneously static and dynamic human molecular imaging. Here, we reported a case of lymph node metastasis from an unknown primary tumor, and the primary tumor was detected with the aid of the total-body PET/CT scanner. This patient with right neck mass was subjected to static and dynamic PET scan, as the static PET imaging found irregular thickening of the upper rectal wall and the dynamic PET imaging recognized the associations between the lymph metastasis and the rectal tumor lesions. The diagnosis by the total-body PET/CT was confirmed by pathological examination.

## Introduction

Metastases is the leading cause of cancer mortality, yet few effective therapeutics are available to combat metastatic lesions (1), as existing therapeutics are predominantly specific to the primary tumor (2). Thus, localization of the primary tumor site is of clinical importance. Strikingly, the lymphatic system stands for the prominent route for tumor cells to metastasize due to much less oxidative stress in lymph relative to that in blood (3). Thereby, metastatic loci are often formed in the lymphatic system prior to the localization in other distant organs (4). In this circumstance, detection of primary tumors with lymph node metastases is of critical importance to understand the likely tumor progression stage and malignancy (5). However, the clinicians are puzzled by the earlier finding of a lymph node metastasis without a certain diagnosis of the primary tumor (6). In other words, lymphatic metastatic tumors sometimes develop before the primary tumor, give rise to perceivable symptoms, develop to a palpable size, or yield meaningful mass of specific cancer biomarkers to be detected.

Clinicians usually look for the primary tumors in the nearby organs using modern imaging modalities. However, the existing imaging modalities including computed tomography (CT) and magnetic resonance imaging (MRI) cannot give a quantitative description of tumor malignancy (7, 8). It is worth mentioning that standardized uptake value (SUV) and its variants from positron emission tomography (PET) can provide semiquantitative analysis of tumor malignancy (7, 8). Thus, PET can be used to look for the primary tumors clinically. However, the searching is limited by inadequate axial field of view (FOV) to locate distant tumor origins (9, 10). Moreover, the diagnosis often suffers from insufficient sensitivities to define small/micro tumors or tumors in latency status (11). Hence, more informative imaging technology is enthusiastically pursued. Under this setting, the recently developed total-body PET/CT system by the EXPLORER consortium represents a state-of-the-art imaging approach to detect all possible tumor sites in the body rapidly and simultaneously and to track the metastatic route (9–11). Here, we report an unusual case with the aid of the total-body PET/CT to look for the unknown primary tumor.

## Case Representation

A 76-year-old male patient was hospitalized with a right neck mass lasting for about 1 month. The patient gave written informed consent for this case report. The patient did not have major trauma, history of surgery, history of blood transfusion, and history of drug and food allergy. Moreover, he did not have family history of cancer. The mass in the right neck was approximately 6 × 5 cm^2^ in size, hard in texture, and absent of tenderness. Conventional body examination did not find any significant abnormalities, and the detection of multiple tumor markers through chemiluminescence immunoassays suggested possible existence of tumors, but with no specific indication of specific tumor type (**Supplementary Table S1**).

**Figure 1:**
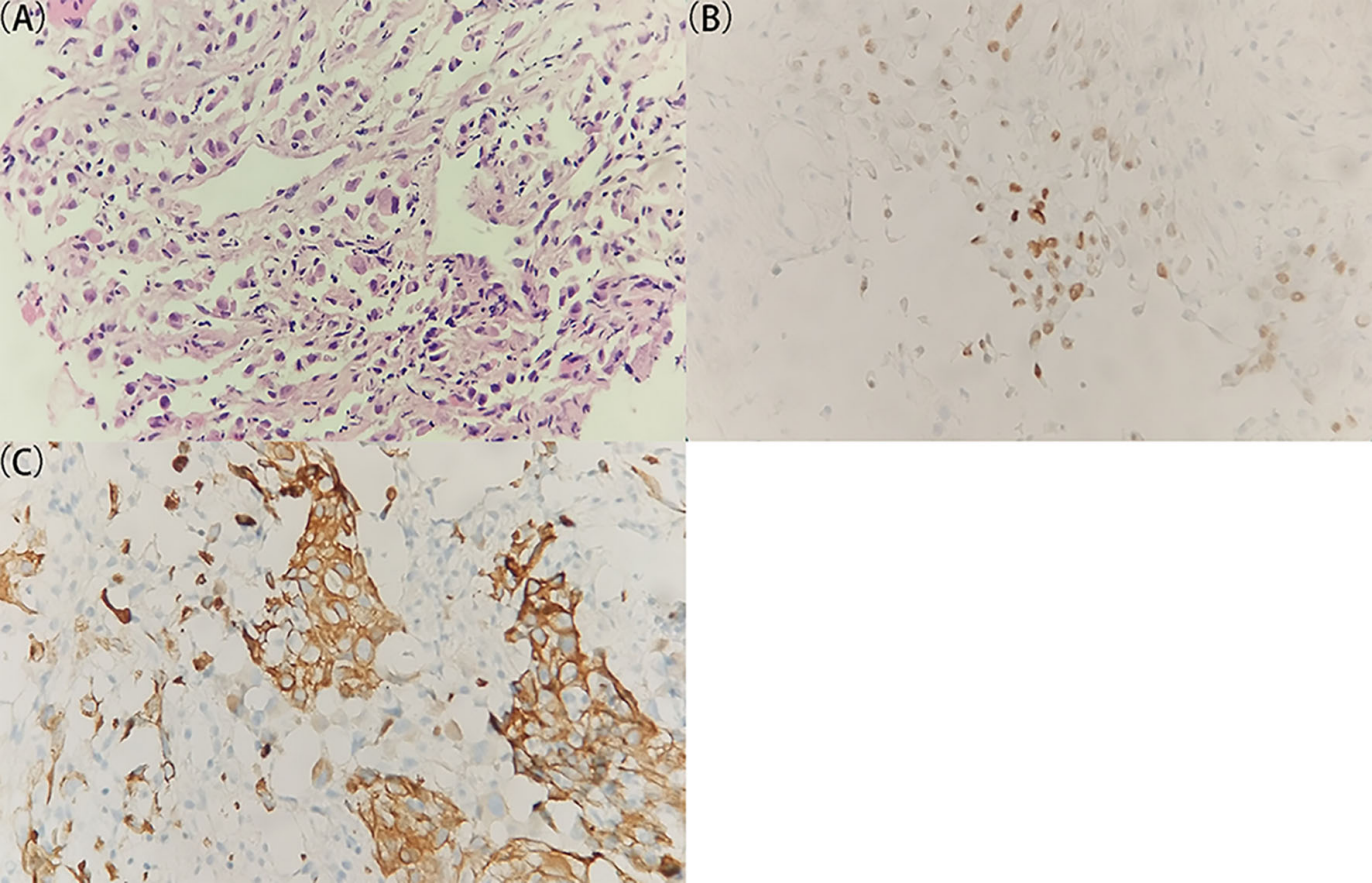
Histological and immunohistochemical examination of the specimen from the right lymph lesion. **(A)** A representative image of the tumor specimen with H&E staining. The original tissue specimen and an H&E staining image with ×40 original magnification are provided in **Supplementary Figure S2**. **(B, C)** Representative images of immunohistochemical staining for **(B)** TTF-1 and **(C)** CK7 which demonstrate positive results. All images are ×400 original magnification. The immunohistochemical results indicate a great probability of metastatic adenocarcinoma for the lymph lesion.

Head and neck MRI revealed a space-occupying lesion in the right neck, indicative of the lymph node metastasis from a malignant tumor (**Supplementary Figure S1**). The biopsy tissue specimen was afterward obtained from the right neck lesion, followed by formalin-fixing, paraffin-embedding, and hematoxylin–eosin (H&E) staining (**Figure 1A**). There were two bands of cord-like tissue with 1.7–1.8 cm in length and 0.1 cm in width and with grayish-white color appearance (**Supplementary Figure S2**). Furthermore, immunohistochemical staining was implemented to detect a panel of tumor markers, including thyroid transcription factor-1 (TTF-1), cytokeratin 7 (CK7), cytokeratin 20 (CK20), caudal type homeobox 2 (CDX2), Villin, prostate-specific antigen (PSA), Napsin A, cluster of differentiation 117 (CD117), S-lfln protein 100 (S-100), thyroglobulin (Tg), tumor protein 63 (p63), Ck5/6, alpha 1-antichymotrypsin (AACT), and alpha-1-antitrypsin (AAT). Moreover, the immunohistochemistry results manifested positive staining for TTF-1 and CK7 (**Figures 1B, C**) and weak positive for AACT and AAT, and negative staining for CK20, CDX2, Villin, PSA, Napsin A, CD117, S-100, Tg, p63, and Ck5/6 in the right neck lesion. These results together hinted a great probability of metastatic adenocarcinoma for the lymph lesion. At this stage, still no perceivable indications of symptoms were identified based on the enquiry, and no cues on a palpable size of tumor were found. Even though lung adenocarcinoma was suspected, lung CT examination revealed no detectable lesion in the bilateral lungs (**Supplementary Figure S3**). To this end, the patient was subjected to the total-body PET/CT scan.

### Dynamic and Static PET/CT Scan

A 1-h dynamic PET scan was performed using the uEXPLORER (United Imaging Healthcare, Shanghai, China) immediately after an intravenous injection of 18F-fluorodeoxyglucose (18F-FDG) according to the patient’s body weight (4.4 MBq/kg) *via* a vein near the ankle after fasting for more than 6 h. Thereafter, the 1-h dynamic PET data were reconstructed using a time-of-flight (TOF) list-mode iterative reconstruction method, as described previously (11). The first 710 s of the 1-h PET data was divided as a sequence of images describing average activity concentrations during a series of time frames, namely, 30 × 1, 20 × 5, 20 × 10, and 19 × 20 s. In the following, 50 min after injection, static total-body PET scan was performed *via* uEXPLORER for 600 s. The PET images were reconstructed using all 600-s data, TOF and point spread function (PSF) modeling, 2 iterations and 20 subsets, matrix = 192 × 192, slice thickness = 2.89 mm, pixel size = 3.125 × 3.125 × 2.886 mm^3^ with a Gaussian filter (FWHM = 3 mm), and all necessary corrections including attenuation and scatter correction. SUV was calculated from the concentration of the radiotracer normalized by injected dose and body weight based on the static PET images, as reported previously (7, 11). Regions with abnormal 18F-FDG uptake were identified and drawn by an experienced radiologist as the volumes of interest. In terms of dynamic PET images, a rigid-body registration was performed to reduce motions among frames. Afterward, the *in vivo* 18F-FDG uptake was quantified as decay-corrected maximum temporal concentration (Bq/ml) in the manually drawn volumes of interest within the dynamic PET images.

### Abnormal 18F-FDG Uptake in the Upper Rectum

**Figure 2:**
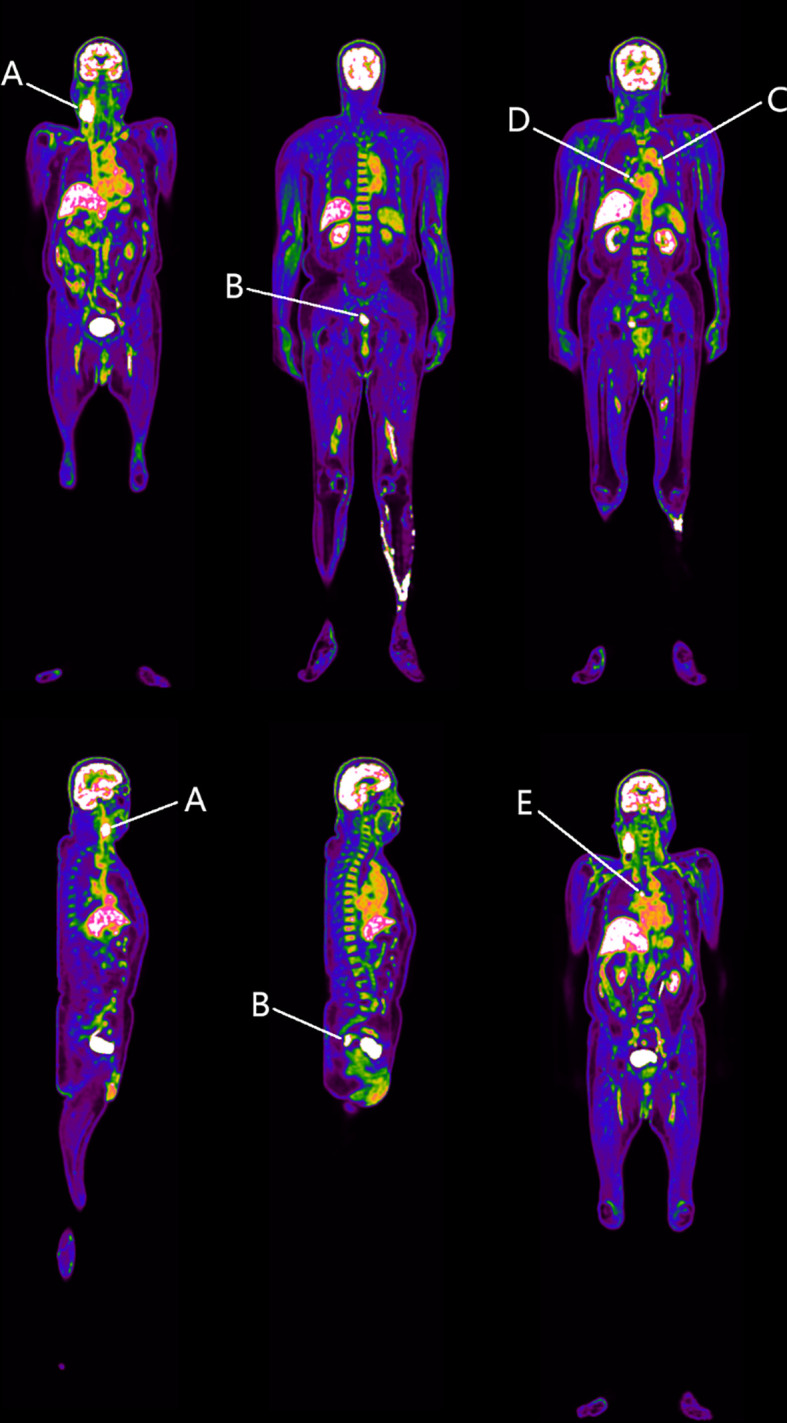
Identified regions with increased 18F-FDG uptake. Region A represents the right neck space-occupying lesion. Region B resides in the rectosigmoid junction. Regions C–E denote the mediastinal lymph nodes. At this stage, we speculate that Region B is the possible primary tumor responsible for Region A.

First, the static total-body PET identified a marked increase in 18F-FDG uptake within the right neck mass with swollen lymph nodes (SUVmax = 21.0) (Region A in **Figure 2**), consistent with metastatic tumor manifestations (12). Strikingly, abnormal 18F-FDG uptake was observed in the upper rectal wall (SUVmax = 12.5), with irregular thickening (Region B in **Figure 2**). However, no similar enhanced 18F-FDG uptake was found in other organs, except multiple lymph nodes in the chest with slightly increased 18F-FDG uptake (SUVmax = 5.1–11.9) (Regions C, D, and E in **Figure 2**). Given that no other sites with abnormal 18F-FDG uptake were observed, a rectal cancer was considered as the possible primary tumor responsible for the lymph node metastasis.

### Association of 18F-FDG Uptake Between the Right Neck Lesion and the Rectal Site

**Figure 3:**
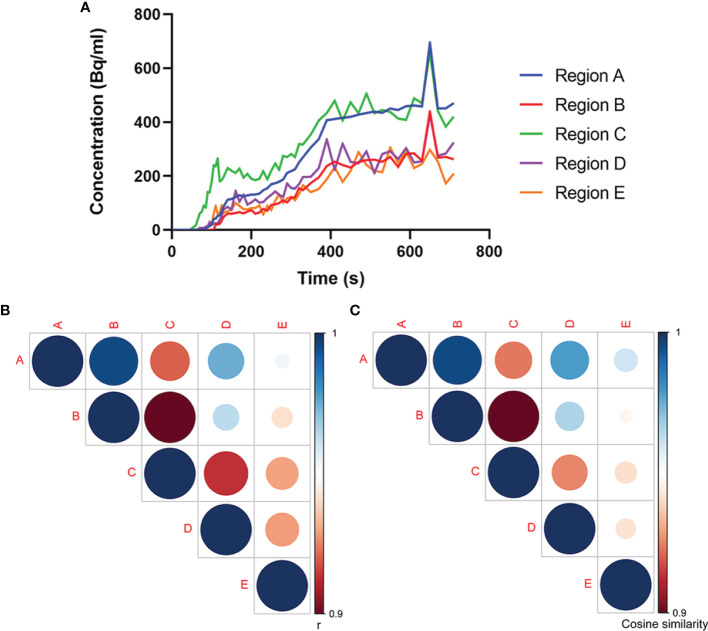
Association analysis of 18F-FDG uptake in multiple regions. **(A)** Time–activity curves of 18F-FDG uptake at different sites. Analyses of **(B)** Pearson’s correlation coefficient and **(C)** cosine similarity index for time–activity curves between every two regions. The association between Regions A and B experiences highest Pearson’s correlation coefficient and cosine similarity index among the associations between every two regions, which confirms our speculation that Region B is the possible primary tumor site for Region A.

The dynamic PET scan was conducted immediately after the injection of 18F-FDG. Regions A (the neck mass in the lymph nodes) and B (the rectal site) showed reinforced 18F-FDG uptake with little fluctuations in the time–activity curves of radiotracers over the time course, presumably due to the pathological uptake. In addition, time–activity curves of Regions A and B manifested a similar trend, with gradually increased 18F-FDG uptake with a marked peak around 650 s. By contrast, dynamic image evaluation revealed greater fluctuations in the regions from C to E in the time–activity curves of 18F-FDG over time (**Figure 3A**).

Further, we analyzed the relationship between the time–activity curves among these five regions using the Pearson’s correlation coefficient and the cosine similarity index. As shown by the Pearson’s correlation coefficient and cosine similarity index (**Figures 3B, C**), among the four regions with abnormal 18F-FDG uptake, Region B displayed the closest relationship toward Region A, suggesting a great similarity between Regions A and B. In this premise, it would be speculated that the rectal tumor was the primary tumor responsible for the right neck lymph node metastasis.

### Enteroscopy and the Final Diagnosis

**Figure 4:**
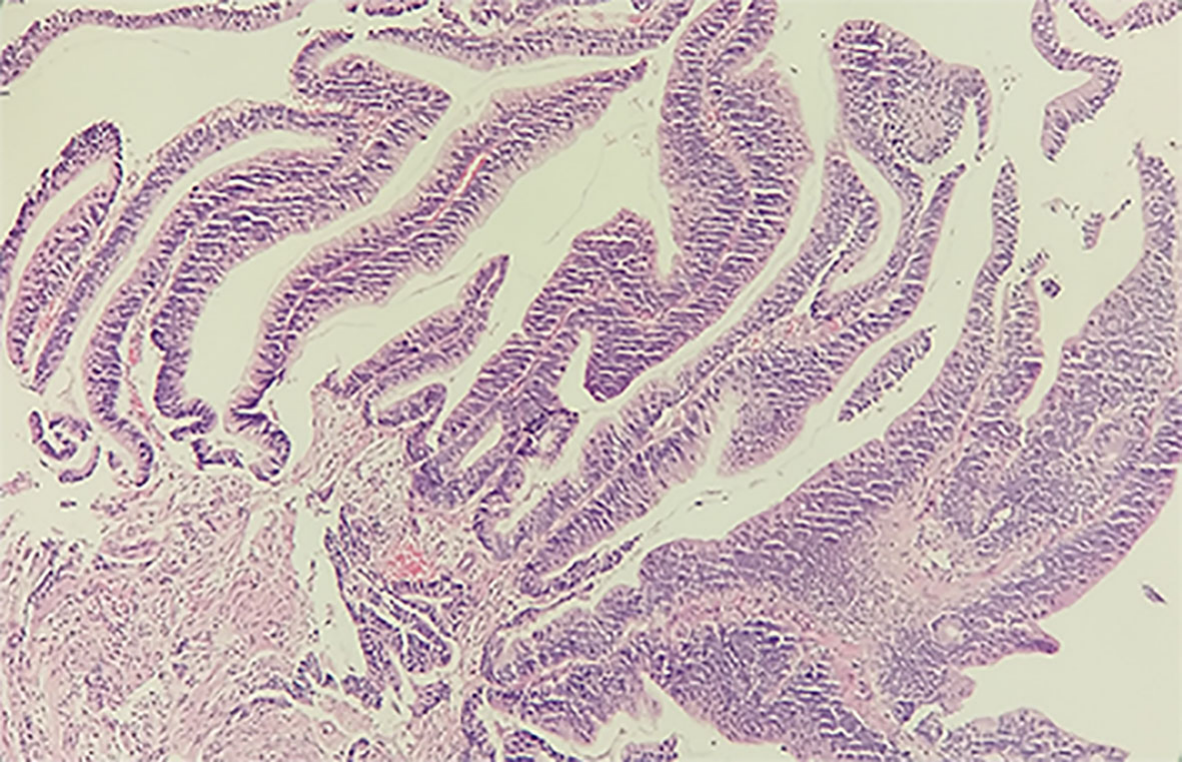
Histological examination of the specimen from the rectosigmoid junction with H&E staining. The tissue is 0.3 × 0.3 × 0.2 cm^3^ in size, grayish-white in color and tough in texture. The high-grade tubulovillous adenoma in the rectosigmoid junction supports the PET/CT diagnosis.

Considering the histological and immunohistochemical examination and PET scan, the patient was confirmed lymph node metastasis. Thereafter, a therapeutic regime was developed with a session of chemotherapy (day 1: pemetrexed 1 g, days 1–3: cisplatin 40 mg, day 4: bevacizumab 400 mg) for 12 days. However, in order to corroborate our hypothesis, enteroscopy, and pathological examination were carried out. The result of enteroscopy unveiled a marked lesion around the rectosigmoid junction, and the biopsy was thus implemented. Collected specimen was subjected to pathological examination, and the histological results recognized a high-grade tubulovillous adenoma (**Figure 4**) with positive staining for CK7, TTF-1, and Napsin A and weak positive staining for Villin. To this end, the pathological results substantiated the PET/CT imaging findings. Eventually, the right neck mass was diagnosed with lymph node metastasis from an adenocarcinoma.

## Discussion

Here, we reported the diagnosis of a patient with lymph node metastasis from an unknown primary tumor using the state-of-the-art total-body PET/CT examination. With the aid of the total-body PET/CT, it became feasible to localize lesions with abnormal 18F-FDG uptake across the whole body, and to tease out the associations between distant tumors and metastatic tumors in the whole body.

Lymph node represents a common site for tumor metastasis, as the lymph node is often the first place for cancer cell to spread in the body and cancer cells experienced less oxidative stress in the lymph relative to blood (3, 4). Under this circumstance, tumor cells often cross the lymphatic wall and circulate through the lymphatic system to reside in the lymph node (13). However, the detection of lymph metastasis may precede the finding of primary tumor, giving rise to an obstacle in localizing tumor origin. Nonetheless, the diagnosis of the primary tumor is of clinical importance in cancer therapeutics, since the site of the primary tumor has a vital role in guiding the clinical care of patients with metastatic tumors (2). Clinicians usually search the nearby organs for possible localization of the primary tumor (14). In the present, the recognition of a distant primary tumor is sometimes impeded by the limitations of the existing imaging modalities, in that the existing imaging systems usually focus on a part of the body and one-bed position. Furthermore, the total-body scan requires multi-bed and multi-pass approaches, with limited temporal resolution and sensitivity due to bed motion (15). Differently, the uEXPLORER total-body PET/CT scanner largely conquers these drawbacks and significantly improves sensitivity (9, 15). In addition to elevated sensitivity and sub-second imaging, the uEXPLORER PET/CT platform collects information on all organs and systems across the whole body simultaneously due to its 194-cm axial FOV (10, 16). Thus far, a few probes have been developed for the imaging purpose on the PET/CT scanner, such as 18F-FDG, 68GA-chloride, and 11C-acetate (17). In this report, we used 18F-FDG-labeled probes for the detection of the primary tumor in a patient with a right neck lymph metastasis on the uEXPLORER PET/CT platform. Our results unearthed an outstanding capacity to identify all abnormal sites through the dynamic and static total-body PET/CT imaging.

Since the primary tumor and the metastatic tumor stem from the same origin and share very similar metabolic features, the 18F-FDG (a glucose analogue) uptake would be nearly constant between the primary tumor and the metastatic tumor. However, it can be rather difficult to simultaneously identify the association of 18F-FDG metabolism among various organs under a static PET/CT imaging (12). Furthermore, although the existing PET scanner with delayed multi-bed scan may hold a chance in discovering the rectal lesion, the existing PET cannot simultaneously detect and show 18F-FDG uptake kinetics across the entire body. Instead, dynamic PET imaging by the uEXPLORER can provide simultaneous spatiotemporal distribution of radiotracers across the entire human body. Therefore, the dynamic PET imaging adds more diagnostic value to the analysis of the static images. A recent study has documented that the dynamic total-body PET imaging enabled the differentiation of pathological 18F-FDG uptake from the physiological uptake based on the visual changes of the uptake profiles (12). Analogously, in the current report, the application of dynamic total-body PET imaging unraveled remarkable discrepancies in the time–activity curves among different regions with abnormal uptake, offering an optimal option to discern the pathological uptake of 18F-FDG from that of physiological uptake at divergent sites. Importantly, the dynamic total-body PET imaging distinguished the similarity in the time–activity curves of 18F-FDG uptake between the right neck mass and the tumor in the rectosigmoid junction, opening a path to locate the primary tumor.

Several concerns need to be addressed in the current report. Firstly, although 18F-FDG is the most common radiotracer in PET imaging. The uptake of 18F-FDG is not specific to cancer and thus may limit the clinical interpretation of the current static and dynamic PET imaging result. A radiotracer targeted for a specific type of tumor may add more diagnostic value. Secondly, the dynamic PET analysis is inevitably interfered by patient’s movement although a rigid-body calibration has been done.

## Conclusion

Overall, the definitive diagnosis of this patient with lymph node metastasis from an unknown primary tumor benefits from the application of the uEXPLORER total-body PET/CT platform in several aspects. On the one hand, the broad axial FOV enables tracking the tracer distribution throughout the whole body simultaneously, which helps localize several potential primary tumor sites with similar abnormal 18F-FDG uptake fingerprints. On the other hand, the dynamic imaging also provides the spatial–temporal information of 18F-FDG uptake throughout the entire body, which not only adds sufficient value to the differentiation of pathological uptake from physiological uptake in lymph nodes but also gives rise to cues on finding associations between distant sites through comparison of 18F-FDG metabolic profiles. Together, we concluded that the total-body PET imaging stands for a robust tool to localize small/micro tumors or tumors in latency across the whole body, and to look for the primary tumor after the finding of lymph metastasis and vice versa. Although this case report provides proof of concept, validation in larger patient cohorts and clinical trials would be essential.

## Data Availability Statement

The raw data supporting the conclusions of this article will be made available by the authors, without undue reservation.

## Ethics Statement

The studies involving human participants were reviewed and approved by the Institutional Review Board of Shandong First Medical University. The patients/participants provided their written informed consent to participate in this study.

## Author Contributions

WL wrote the manuscript. JQ and XX performed image preprocessing and statistical. KL, YD, ML, and CM preformed image acquisition and prepared clinical data. ZC and SL reviewed the manuscript. All authors contributed to the article and approved the submitted version.

## Funding

This work was supported by the “Outstanding University Driven by Talents” Program and Academic Promotion Program of Shandong First Medical University (grant number: 2020LJ002, 2019QL009), Science and Technology Funding from Jinan (grant number: 2020GXRC018), and the Taishan Scholars Program of Shandong Province (grant number: TS201712065).

## Conflict of Interest

The authors declare that the research was conducted in the absence of any commercial or financial relationships that could be construed as a potential conflict of interest.

## Publisher’s Note

All claims expressed in this article are solely those of the authors and do not necessarily represent those of their affiliated organizations, or those of the publisher, the editors and the reviewers. Any product that may be evaluated in this article, or claim that may be made by its manufacturer, is not guaranteed or endorsed by the publisher.
