# Supplementary material for: Recognizing Tumor Origin for Lymphoid Tumor of Unknown Primary via Total-Body PET/CT Scan—Case Report
Source: Front Oncol. 2022 Feb 3;12:766490. doi: 10.3389/fonc.2022.766490 (PMC8850463; doi:10.3389/fonc.2022.766490)
Supplement: Supplementary file 1 [file DataSheet_1.docx]

Supplementary Material

# Supplementary Table S1. Conventional examination indexes and the serum levels of tumor markers.

| **Variable** | | **Result** | **Reference** |
| --- | --- | --- | --- |
| Physical examination | Body temperature | 36.2°C | - |
|  | Heart rate | 87 beats/min | - |
|  | Respiratory rate | 20 breaths/min | - |
|  | Blood pressure | 156/103 mmHg | - |
| Serum  tumor markers | Carcinoembryonic antigen | 5.32 ng/mL | 0-5 ng/mL |
|  | Total prostate specific antigen | 4.203 ug/L | 0-4 ug/L |
|  | Free prostate specific antigen | 1.546 ug/L | 0-1 ug/L |
|  | Neuron-specific enolase | 17.79 ng/mL | 0-16.3 ng/mL |
|  | Cytokeratin-19-fragment CYFRA21-1 | 6.46 ng/mL | < 3.3 ng/mL |
|  | Squamous cell carcinoma antigen | 6.0 ng/mL | < 1.5 ng/mL |
|  | [Carbohydrate antigen](http://www.baidu.com/link?url=NXOU_kshVbz-ZRI2Xr8L-8y2UV0OxFRh8sSGMW61jMl0GVFlFO2qd7noKNbG6aAd9EjiW752Y7VyB6qiYLemw_r-w05-u3zcMtrAs0MYH1Lii8I6uDK3Mk4jI6TofQgK)-50 | 34.27 IU/mL | 0-25 IU/mL |


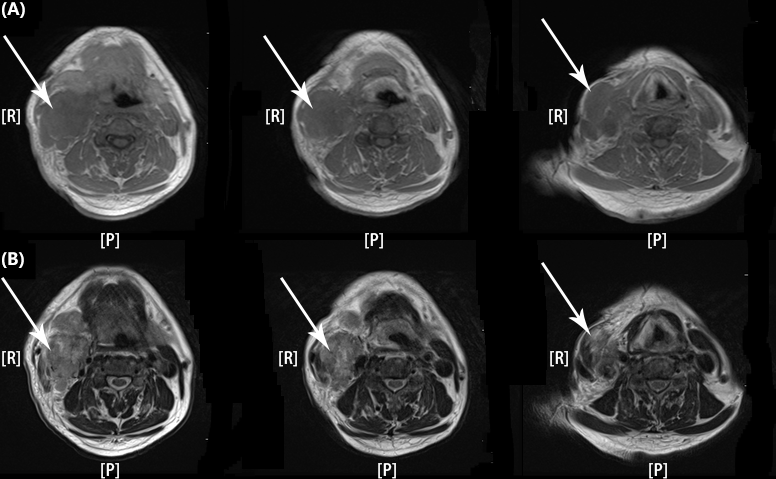


**Supplementary Figure 1.** **The head and neck MRI images of the patient.** (A) T1-contrasted images with flip angle (FA) = 70°, time of repetition (TR) = 250 ms, echo time (TE) = 2.46 ms. The three T1 contrasted images are the 16^th^, 18^th^ and 20^th^ slices in the axial orientation. (B) T2-weighted images with FA = 180°, TR = 5260 ms, TE = 83 ms. The three T2-weighted images are the 16^th^, 18^th^ and 20^th^ slices in the axial orientation. R: right, P: posterior. The arrow marks a space-occupying lesion in the right neck, indicating a lymph node metastasis from a malignant tumor.


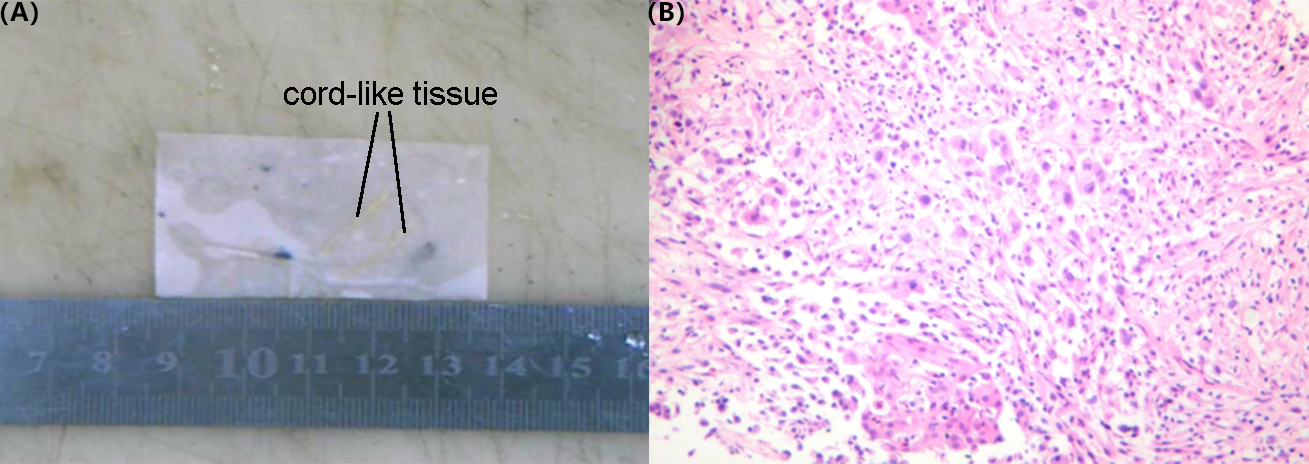


**Supplementary Figure 2.** **Histological examination of the specimens from the right lymph lesion**. (A) An image of the lymph specimen with two bands of cord-like tissue. (B) A representative image of the specimen with H&E staining.

**
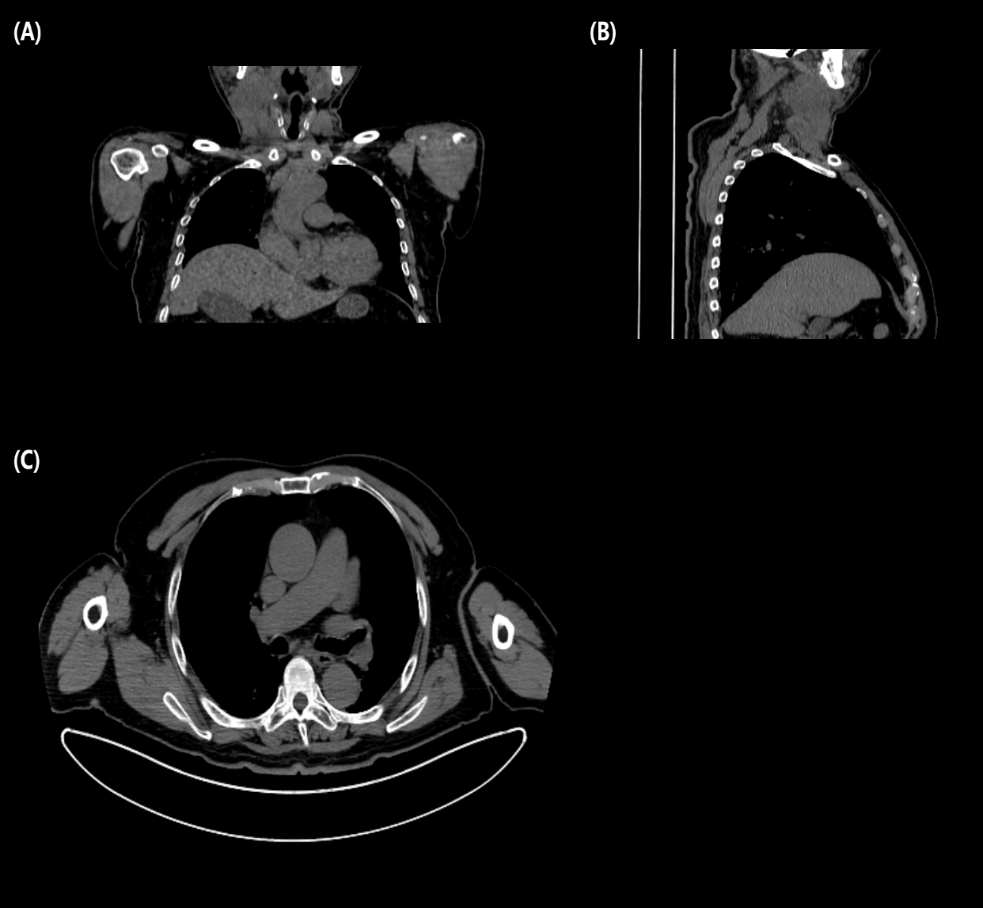
**

**Supplementary Figure 3. The lung CT image of the patient.** (A) Coronal view. (B) Sagittal view. (C) Axial view. Although lung adenocarcinoma is firstly suspected as the primary tumor site for the lymph lesion, lung CT examination reveals no detectable lesion in the bilateral lungs. At this stage, the patient is subjected PET/CT scan to search for possible primary tumor.
